# Magnetic susceptibility changes in the brainstem reflect REM sleep without atonia severity in isolated REM sleep behavior disorder

**DOI:** 10.1038/s41531-023-00557-2

**Published:** 2023-07-14

**Authors:** Jiri Nepozitek, Zsoka Varga, Simona Dostalova, Pavla Perinova, Jiri Keller, Simon Robinson, Veronika Ibarburu, Iva Prihodova, Ondrej Bezdicek, Evzen Ruzicka, Karel Sonka, Petr Dusek

**Affiliations:** 1 Department of Neurology and Center of Clinical Neuroscience, First Faculty of Medicine, Charles University and General University Hospital in Prague, Prague, Czech Republic; 2 Radiodiagnostic Department, Na Homolce Hospital, Prague, Czech Republic; 3 Department of Biomedical Imaging and Image-guided Therapy, Medical University of Vienna, Vienna, Austria; 4 Centre of Advanced Imaging, University of Queensland, Brisbane, Queensland Australia

**Keywords:** Neurodegeneration, Diagnostic markers

## Abstract

REM sleep without atonia (RWA) is the hallmark of isolated REM sleep behavior disorder (iRBD) and is caused by neurodegeneration of brainstem structures. Previously, quantitative susceptibility mapping (QSM) was shown to detect microstructural tissue changes in neurodegenerative diseases. The goal of the study was to compare brainstem magnetic susceptibility (MS) in iRBD and controls using the voxel-based QSM approach and to examine the association between brainstem MS and severity of RWA in iRBD. Sixty iRBD patients and 41 healthy controls were included in the study. Phasic, tonic, mixed RWA and SINBAR score was quantified. QSM maps were reconstructed with QSMbox software from a multi-gradient-echo sequence acquired at 3T MRI system and normalized using a custom T1 template. Voxel-based analysis with age and gender as covariates was performed using a two-sample t-test model for between-group comparison and using a linear regression model for association with the RWA parameters. Statistical maps were generated using threshold free cluster enhancement with *p*-value *p* < 0.05, corrected for family wise error. Compared to controls, the iRBD group had higher MS in bilateral substantia nigra (SN), red nucleus and the ventral tegmental area. MS positively correlated with iRBD duration in the right pedunculotegmental nucleus and white matter of caudal mesencephalic and pontine tegmentum and with phasic RWA in bilateral SN. QSM was able to detect MS abnormalities in several brainstem structures in iRBD. Association of MS levels in the brainstem with the intensity of RWA suggests that increased iron content in SN is related to RWA severity.

## Introduction

Rapid eye movement (REM) sleep behavior disorder (RBD) is a parasomnia, characterized by abnormal behavior during rapid eye movement (REM) sleep corresponding to the current dream content^1,2^. Isolated RBD (iRBD) is considered as an early manifestation of neurodegenerative diseases associated with alpha-synuclein aggregation, namely Parkinson’s disease (PD), dementia with Lewy bodies (LBD) and multiple system atrophy (MSA)^3–7^. Longitudinal studies have demonstrated that most iRBD patients will eventually develop one of the synucleinopathy phenotypes^8^. Diagnostics of these neurodegenerative disorders in the stage of iRBD provide an opportunity to search for potential prodromal markers that may serve to follow the disease’s progression, predict the phenoconversion to overt synucleinopathy and hypothetically to assess the treatment response, once potentially neuroprotective therapy becomes available^9^.

Dream enactment behavior in iRBD results from the loss of the normal skeletal muscle atonia during REM sleep which is noticeable as increased electromyographic (EMG) activity during polysomnography and referred to as REM sleep without atonia (RWA). Based on their temporal characteristics, tonic and phasic types of RWA are distinguished^10,11^. The Sleep Innsbruck Barcelona (SINBAR) group tested multiple muscle montages and reported that the best approach for diagnosis of RWA is a combination of any EMG activity in the chin and/or phasic EMG activity in the flexor digitorum superficialis (FDS) muscle, thus SINBAR score was suggested as a quantification tool^12^. Recently another quantifiable parameter of RWA distinct from previously described tonic and phasic activity was defined; it is characterized by the simultaneous occurrence of phasic and tonic activity and is called mixed RWA^13^.

Several observational and longitudinal studies have documented that RWA may serve not only as a diagnostic marker but also as a marker of disease progression and impeding phenoconversion^5,14–18^. It was suggested that RWA intensity may be used as a quantifiable biomarker reflecting the severity of neurodegenerative changes in iRBD^13,19–22^.

RWA and dream enactment are supposedly caused by the involvement of brainstem structures^23^. Studies investigating the neuropathology of RBD have demonstrated that increased RWA activity results from the degeneration of the sublateralis dorsalis tegmenti (SLD, nucleus subcoeruleus)^24–26^, which 1) makes spinal ventral horn interneurons unable to inhibit spinal motor neurons and 2) disrupts inhibitory circuits that project to the ventral-medial medullary reticular formation (VMM), causing the loss of its ability to directly inhibit spinal motor neurons^27–29^. In addition to the involvement of SLD and VMM, other brainstem areas such as substantia nigra (SN) – pars compacta and pars reticulata, pedunculotegmental nucleus (PTg, also known as pedunculopontine nucleus), raphe nuclei, dorsal motor nucleus of the vagal nerve, cuneiform nucleus and periaqueductal gray were shown to be affected by the neurodegenerative process at the disease stage manifested as iRBD^30,31^.

Over the past years, an increasing number of studies have been searching for neuroimaging markers depicting neurodegenerative brain changes in iRBD^32,33^. Neurodegenerative processes of the human brain were found to be associated with increased brain iron deposition and microstructural changes such as loss of neurons, myelinated tracts and reactive gliosis. All of these changes may alter the magnetic properties of tissues which can be detected by MRI^34^. Among others, magnetic resonance imaging (MRI) techniques measuring iron concentration were proposed to be sensitive for the detection of neurodegenerative changes^35^. The overload of paramagnetic iron species, visible as hypointense changes on susceptibility-weighted imaging (SWI), can be semi-quantified using R2* relaxometry or quantitative susceptibility mapping (QSM)^36–38^ whereby QSM showed a higher sensitivity for the differentiation of PD vs. controls compared to R2* relaxometry^39^. Increased iron content in SN has previously been reported in PD and DLB^40–44^, i.e., when symptoms of nigrostriatal degeneration are clinically apparent. There are only a few studies on brain iron deposition in iRBD and their results are inconsistent. The first study that looked for the brain iron content in iRBD found no alterations using transverse relaxation rate (R2*) assessment^45^. Later, abnormalities in SN suggestive of iron deposits were detected in iRBD using SWI^46^ and QSM^47^. A recent QSM study showed that although no difference between iRBD and healthy individuals was found, a positive correlation was noted between magnetic susceptibility in the nigrosome 1 and disease duration^48^.

Given that iRBD is a manifestation of a neurodegenerative process that is known to affect many brainstem regions, it is reasonable to expect, that QSM, which is sensitive to the iron content and other microstructural changes affecting tissue magnetic properties, may reveal brainstem changes related to neurodegeneration in iRBD. The recognition that REM sleep atonia loss progresses over time in iRBD suggests that RWA can be a suitable reference parameter of clinical disease severity for the investigation of the magnetic susceptibility changes associated with neurodegeneration. This study aims to compare brainstem magnetic susceptibility changes in iRBD and healthy controls and to test the hypothesis that magnetic susceptibility changes in the brainstem correspond to the severity of RWA.

## Results

### Characteristics of study participants

The overview of demographic, clinical and polysomnographic parameters is presented in Table 1. Patients with iRBD had higher RBD screening questionnaire (RBD SQ) and Movement Disorders Society-sponsored Revision of the Unified Parkinson’s Disease Rating Scale (MDS-UPDRS) part III scores, sleep latency, periodic limb movements index (PLMI) and all RWA indices, and lower Montreal Cognitive Assessment (MoCA) scores, and apnea-hypopnea index (AHI) compared to controls.

**Table 1:** Demographic, clinical and sleep parameters.

	iRBD (*n* = 60)		Controls (*n* = 41)		*p*
	Mean	SD	Mean	SD	
Age [years]	67.0	7.2	63.1	9.7	0.051
Gender (F/M)	6/54		10/31		0.094^a^
EHI (R/N)	58/2		37/4		0.220^a^
RBD duration [years]	6.7	5.5	NA	NA	NA
RBD SQ	9.6	2.3	1.6	1.4	<0.001*
MDS-UPDRS III	5.7	5.2	3.7	4.1	**0.018**
MoCA	23.6	3.1	25.0	2.4	**0.005**
Polysomnography					
Sleep efficiency [%]	75.6	12.3	72.2	16.8	0.483
TST [min.]	339.3	60.8	320.7	77.5	0.349
Sleep latency [min.]	22.6	18.9	15.7	13.2	**0.020**
REM latency [min.]	111.3	72.9	110.2	64.0	0.809
W [%]	19.9	11.4	25.2	16.1	0.154
N1 [%]	11.2	11.9	9.3	5.1	0.491
N2 [%]	38.7	11.0	34.8	10.9	0.127
N3 [%]	15.4	7.3	16.3	7.5	0.326
REM [%]	16.3	5.7	14.3	7.3	0.151
AHI	11.1	12.3	19.8	17.1	0.001*
PLMI	26.9	34.0	12.5	22.5	**0.005**
Arousal index	14.5	8.4	18.9	10.0	**0.006**
RWA					
SINBAR score [% REM]	48.6	21.4	5.6	4.2	<0.001*
Phasic RWA [% REM]	25.0	12.6	3.4	2.9	<0.001*
Tonic RWA [% REM]	16.8	15.7	1.0	1.1	<0.001*
Mixed RWA [% REM]	7.9	7.8	0.3	0.5	<0.001*

*F* females, *M* males, *EHI* Edinburgh handedness inventory, *R* right-handedness, *N* non-right-handedness, *RBD* rapid eye movement sleep behavior disorder, *RBD SQ* rapid eye movement sleep behavior disorder screening questionnaire, *MDS-UPDRS III* Movement Disorders Society-sponsored Revision of the Unified Parkinson’s Disease Rating Scale, part III, *MoCA* Montreal Cognitive Assessment, *TST* total sleep time, *min.* minutes, *W* wakefulness, *N1, 2, 3* non-rapid eye movement sleep stage 1, 2, 3, *REM* rapid eye movement sleep, *AHI* apnea-hypopnea index, *PLMI* periodic limb movements index, *RWA* rapid eye movement sleep without atonia, *SINBAR* Sleep Innsbruck Barcelona, *iRBD* isolated rapid eye movement sleep behavior disorder, *NA* not applicable, *SD* standard deviation.

Bold values indicates *p*-values < 0.05, *Remains significant after Holm–Bonferroni correction (20 tests applied).

^a^Fisher’s exact two-tailed applied.

### Comparison of the susceptibility between iRBD and controls

**Fig. 1:**
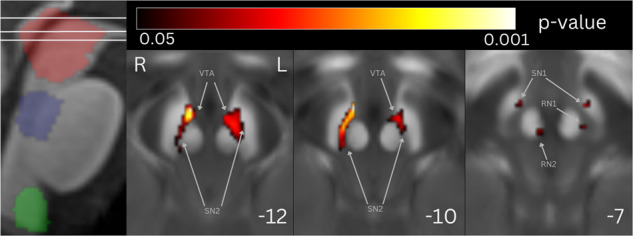
Statistical map of voxel-wise QSM analysis showing the contrast iRBD > controls. The statistical map is overlayed onto the study-wise QSM template transformed to the MNI coordinate system thresholded at *P*_FWE_ < 0.05 at cluster level. Z-coordinates in mm and the respective position on sagittal T1 image are shown for each slice. Mesencephalic/ponto-mesencephalic border (red), dorsal pontine (blue) and ventral medullary (green) mask projections are shown at the sagittal T1 image. Elevated magnetic susceptibility can be seen in bilateral SN (mostly pars compacta), RN and VTA. VTA ventral tegmental area, SN1 substantia nigra pars reticulata, SN2 substantia nigra pars compacta, RN1 red nucleus – subregion 1, RN2 red nucleus – subregion 2.

Compared to controls, the iRBD group had higher magnetic susceptibility in bilateral SN (mostly involving pars compacta), RN and ventral tegmental area (VTA) (Fig. 1, Table 2) within the mesencephalic/ponto-mesencephalic border mask. No significant results were found in dorsal pontine and ventral medullary masks.

**Table 2:** Statistic results of cluster-level analysis, coordinates of peak voxel in MNI space, brainstem nuclei involved in the cluster according to Brainstem Navigator.

*k*_E_	*p*_(FWE-corr)_	Coordinates			Involved structures
		X [mm]	Y [mm]	Z [mm]	
iRBD vs. controls					
801	0.010	−7	−17	−12	SN1, SN2, RN1, VTA
682	0.002	8	−15	−11	SN1, SN2, VTA
35	0.037	4	−22	−7	RN2
Disease duration					
93	0.046	4	−27	−18	PTg
Phasic RWA					
285	0.028	−10	−14	−12	SN1, SN2
58	0.042	8	−14	−12	SN1, SN2

*k*_*E*_ expected voxels per cluster, *p*_*(FWE-corr)*_ family wise error-corrected *p*-value, *iRBD* isolated rapid eye movement sleep disorder, *RWA* rapid eye movement sleep without atonia, *PTg* pedunculotegmental nucleus, *VTA* ventral tegmental area, *SN1* substantia nigra pars reticulata, *SN2* substantia nigra pars compacta, *RN1* red nucleus – subregion 1, *RN2* red nucleus – subregion 2.

### Correlation of magnetic susceptibility with disease duration and MDS-UPDRS III

**Fig. 2:**
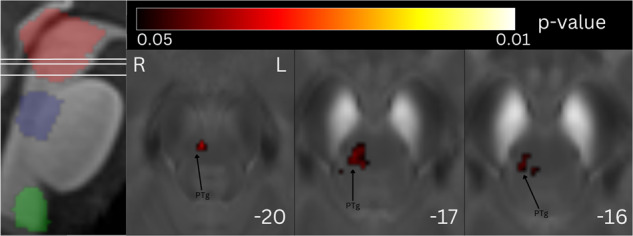
Statistical map of voxel-wise QSM analysis showing the correlation between susceptibility and disease duration. The statistical map is overlayed onto the study-wise QSM template transformed to the MNI coordinate system, thresholded at *P*_FWE_ < 0.05 at cluster level. Z-coordinates in mm and the respective position on sagittal T1 image are shown for each slice. Mesencephalic/ponto-mesencephalic border (red), dorsal pontine (blue) and ventral medullary (green) mask projections are shown at the sagittal T1 image. The involvement of right PTg and white matter of caudal mesencephalic and pontine tegmentum can be seen. PTg pedunculotegmental nucleus.

For the mesencephalic/ponto-mesencephalic border mask, magnetic susceptibility correlated positively with iRBD duration in right PTg and white matter of caudal mesencephalic and pontine tegmentum, corresponding to the area of right superior cerebellar peduncle fibers (“white nucleus”), right central tegmental tract and right lemniscus medialis (Fig. 2, Table 2). No significant correlation for MDS-UPDRS III was found. No significant results were found in dorsal pontine and ventral medullary masks.

### Correlation of magnetic susceptibility and RWA

**Fig. 3:**
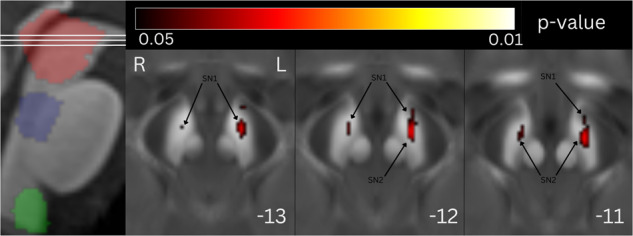
Statistical map of voxel-wise QSM analysis showing the correlation between susceptibility and phasic RWA. The statistical map is overlayed onto the study-wise QSM template transformed to the MNI coordinate system, thresholded at *P*_FWE_ < 0.05 at cluster level. Z-coordinates in mm and the respective position on sagittal T1 image are shown for each slice. Mesencephalic/ponto-mesencephalic border (red), dorsal pontine (blue) and ventral medullary (green) mask projections are shown at the sagittal T1 image. The involvement of bilateral SN (including both pars compacta and pars reticulata) can be seen. SN1 substantia nigra pars reticulata, SN2 substantia nigra pars compacta.

For the mesencephalic/ponto-mesencephalic border mask, a positive correlation between magnetic susceptibility and phasic RWA was found in bilateral SN (both pars compacta and pars reticulata) (Fig. 3, Table 2). No significant correlation for SINBAR score, tonic and mixed RWA was found at the current FWE level. No significant results were found in dorsal pontine and ventral medullary masks.

## Discussion

In this study we have analyzed brainstem QSM in iRBD patients and healthy controls and found increased magnetic susceptibility in SN pars compacta, RN and VTA in the iRBD group. Magnetic susceptibility in SN positively correlated with RWA severity and magnetic susceptibility in pontine tegmentum was positively associated with disease duration.

Only few studies have previously assessed magnetic susceptibility in iRBD and their findings are not consistent. While some found no differences from healthy controls^45,48^, others reported increased magnetic susceptibility in SN^46,47^ similarly to our study. Disparate findings are likely related to the method of SN segmentation. In contrast to previous studies, we have performed voxel-wise QSM analysis which allows the detection of changes without the necessity of manual segmentation of nuclei of interest. Our results show that magnetic susceptibility in iRBD is elevated mostly in dorsal and medial SN subregions, consistent with its pars compacta. Manual SN segmentation inevitably leads to the inclusion of SN subregions without susceptibility changes in iRBD and some voxels located in the affected part of SN pars compacta in the segmentation mask are expected to influence these results^49^.

Increased magnetic susceptibility was found in SN pars compacta, RN and VTA regions in iRBD. SN pars compacta and RN are both iron-rich structures with a known propensity for iron accumulation in aging and pathologic conditions^50,51^. While higher iron content in SN in connection to the neurodegenerative process is repeatedly observed, only a few studies mention susceptibility changes in RN in PD. Increased R2* rates in RN were found in PD in relation to compensation mechanisms of levodopa-induced dyskinesia^52^ and RN visualized on SWI showed reduced volume in late-onset PD and a positive association of volume and PD duration^53^. In an attempt to explain our findings, we can speculate that alpha-synuclein pathology could represent an insult, under the condition of which, iron accumulates in several vulnerable areas of the brain as part of the neurodegeneration process. As for VTA, recently there is growing evidence that it plays a role in the regulation of sleep and wakefulness and suppression, induction and maintenance of REM sleep^54,55^. It can be speculated that pathological iron accumulation in VTA could contribute to the RWA manifestation.

We found changes in magnetic susceptibility in right PTg and white matter of the tegmental area in the caudal mesencephalon and pons positively correlating with the duration of the disease. PTg is known to be important for arousal and motor functions^56,57^ and regulation of REM sleep^58^, which is compatible with previous observations of the involvement of PTg associated with RBD^31^. Increased magnetic susceptibility in the white matter can be caused by the loss of myelin^34^, so our finding of white matter involvement can be theoretically interpreted as indirect signs of loss of myelinated fibers due to neurodegenerative changes. The apparent unilateral involvement of right PTg is not surprising since asymmetrical involvement of brain structures is repeatedly reported in neuroimaging studies concerning iRBD^59,60^. Importantly, our findings are in line with previous studies examining associations of magnetic susceptibility with disease duration in iRBD^47,48^.

Surprisingly, although increased signal in PTg was found in correlation with disease duration, it was not present in the analysis comparing iRBD patients vs. controls. This finding is uneasy to interpret. One possible explanation could be that the dynamics of iron deposition differ in individual subregions - in SN the involvement might occur later or more abruptly, while in PTg it is more continuous and gradual, so it better follows the values of the disease duration. However, a major limitation of the conclusion is that disease duration is a value that is reported by patients, so it is burdened with a certain degree of individual subjective inaccuracy and it is possible that it might have influenced the results.

This study is, to our knowledge, the first focusing on the relationship between RWA and brainstem magnetic susceptibility changes in iRBD. We found a positive correlation between magnetic susceptibility and phasic RWA in bilateral SN.

RWA is repeatedly reported as both a diagnostic and a prognostic quantifiable biomarker. Therefore, its association with the level of susceptibility in SN suggests that susceptibility also defines the severity of the disease and thus confirms that it can be considered a biomarker, as already suggested in previous studies^46–48^.

These findings further confirm and extend the results of reports indicating an association between REM sleep muscle activity and SN degeneration detected by DAT-SPECT^21,22,61^. It is not likely that the relation of magnetic susceptibility with RWA results from the direct influence of SN dysfunction on the muscle activity in REM sleep since the regulation of muscle atonia in REM sleep is known to be regulated by different specific structures including SLD and VMM^23–29^. Rather, we hypothesize that in iRBD the alpha-synuclein pathology affects multiple brainstem neuronal structures in parallel, including SN and REM sleep atonia-maintaining neuronal populations. Thus, we might consider abnormal susceptibility levels in SN and RN structures and RWA as two parallel features of ongoing alpha-synucleinopathy.

While a significant positive correlation between magnetic susceptibility in SN and phasic RWA was observed, no correlations with tonic and mixed RWA and SINBAR score were found. It has been suggested that phasic and tonic muscle activity in REM sleep in RBD have different underlying neural mechanisms^14^. Phasic RWA was reported to be generated by cortical and spinal motor neurons^62^ and depends upon the alteration of the activity of VMM^14,23,27^ and tonic RWA is considered to be caused by degeneration of SLD^14,63^. At the same time, however, populations of REM-active neurons in SLD are considered to be generally important for the generation of REM sleep atonia by projection to both inhibitory promotor neurons in the spinal cord and VMM. Mixed RWA was speculated to be an expression of overlapping involvement of several functional subsections of the SLD^13^. Under these assumptions, we can conclude that the finding of a significant correlation between magnetic susceptibility and phasic RWA reflects, predominantly, the involvement of VMM. The present observations are in line with the ascending pathological model indicating that medulla oblongata is affected first in alpha-synuclein pathology^30^.

Surprisingly, susceptibility changes were not detected directly in the areas of expected primary REM sleep atonia-maintaining structures (SLD and VMM). Apparently, although QSM is able to reflect indirect signs of degeneration, it detects increased accumulation of paramagnetic substances (mostly iron) and abnormalities of diamagnetic myelin. These features may not be the same for all neuronal structures, with only some showing the tendency for iron accumulation during the neurodegenerative process.

The finding that the correlation of susceptibility with mixed RWA was not found cannot support the previously formulated notion that mixed RWA is a result of overlapping neurodegeneration involving multiple brainstem areas^13^. Mixed RWA was speculated to be a manifestation of SLD degeneration itself, in which case SLD, as a more rostrally located nucleus, may be affected later than VMM, and therefore we find a lower association with susceptibility compared to phasic RWA, which is already at the level of VMM with earlier degeneration more expressed. If mixed RWA is a reflection of pure “intersection” between SLD and VMM dysfunction, our finding would suggest that VMM and SLD are affected unequally.

Elevated tonic and mixed RWA have previously been observed to be associated with higher phenoconversion risk and their predictive values have been consistently more specific than in phasic RWA^8,13–15^. On the other hand, phasic RWA takes part in the overall RWA evaluation which exerts progression over the course of the disease and has proven to be significant for diagnosis (as part of the SINBAR recommendation)^12,18^. These previously reported features of phasic RWA are consistent with the finding of a positive relationship with susceptibility and are in line with the idea that it could be a manifestation of the earlier periods of prodromal alpha-synucleinopathy.

There are several potential limitations of the study. Firstly, the cohort comprises a relative gender disproportion with a predominance of males, which is in agreement with some previous epidemiological reports^64,65^ and with enrollment in prospective iRBD cohorts^6,66,67^. Also, AHI indices among both patient and control groups were relatively high. We believe that this did not influence the results because RWA episodes associated with respiration events were identified reliably and excluded from the RWA quantification. Even though we did not use all the available subjects for the template creation to make the preprocessing steps less demanding on processing power, we believe this technical choice did not affect the outcome of the analysis in any way. The cross-sectional design of the study could also be considered a limitation, however this study was sufficient to answer the formulated hypotheses and created a basis for future observational or longitudinal research. The strength of the study is a relatively large number of participants; investigation of magnetic susceptibility in iRBD has not yet been performed on such a scale.

In conclusion, QSM was able to detect magnetic susceptibility abnormalities in several brainstem structures in iRBD. The association of magnetic susceptibility levels in the brainstem with the intensity of RWA suggests that increased iron content in SN is related to RWA severity. These results support the role of magnetic susceptibility as a biomarker of alpha-synucleinopathy.

## Methods

### Research participants

The patients were recruited by a stepwise media and internet survey^68^. Sixty consecutive patients were diagnosed with iRBD according to the latest version of the International Classification of Sleep Disorders, third edition (ICSD-3). The diagnosis of RBD was based on their history of dream-enacting behaviors and nocturnal video-polysomnography (vPSG) demonstrating excessive EMG activity during REM sleep^11^. The exclusion criteria were as follows: age under 50 years, clinical signs of overt dementia or parkinsonism, RBD associated with narcolepsy, encephalitis and head injury, or the presence of focal brainstem lesions on MRI indicative of secondary RBD. The control group was recruited through advertisement and consisted of 41 healthy volunteers aged 50 years or more with no medical history of sleep or neurological disorders. In the medical history of the study group, the use of antidepressants and anxiolytics during and prior to the study was specifically documented. Only patients who reported the beginning of RBD symptoms before they started using antidepressants or anxiolytics were included. Fourteen of the iRBD patients were taking antidepressants (two patients were being treated with citalopram, three with escitalopram, two with trazodone, three with sertraline, one with fluoxetine, one with paroxetine, one with venlafaxine and one with bupropione), nine were taking anxiolytics (six patients were being treated with clonazepam and three with alprazolam), three were taking both antidepressants and anxiolytics. For anxiolytics, the evening dose was omitted before PSG. The study was approved by the Ethics Committee of the General University Hospital in Prague and participants signed written informed consent before entering the study, in accordance with the Helsinki Declaration.

### Clinical examination

All study participants underwent a comprehensive protocol consisting of a comprehensive medical history, a neurological examination including the MDS-UPDRS^69^, vPSG, brain MRI and a neuropsychological examination including a clinical interview and the MoCA as a measure of global cognitive function^70^. All participants completed a Czech version of the RBD SQ (0–13 points)^71,72^ and Edinburgh Handedness Inventory (EHI)^73^ interpreted according to the resulting laterality index as right-handedness (>+40) or non-right-handedness (+40 or less) (including ambidexterity: between +40 and −40 and left-handedness: <−40).

### Video-polysomnography

Nocturnal vPSG was performed using a digital polysomnography system (RemLogic, version 3.4.1, Embla Systems) and consisted of electrooculography (EOG), electroencephalography (F3-M2, C3-M2, O1-M2, F4-M1, C4-M1, O2-M1), surface EMG of the bilateral mentalis, FDS and tibialis anterior muscles, electrocardiography, nasal pressure, nasal and oral air flow, thoracic and abdominal respiratory effort, oxygen saturation, microphone and digitally synchronized video monitoring, measured during the period from 10 p.m. to 6 a.m. according to the American Academy of Sleep Medicine (AASM) recommendation^74^. All features on video-PSG were analyzed visually. The sleep stages, arousals, respiratory events and limb movements were scored according to the AASM Manual for the Scoring of Sleep and Associated Events version 2.2 2015^74^ with an exception for the REM sleep rules allowing for the scoring of the REM sleep stage despite the prominent EMG activity in the mentalis muscle channel^75^. The beginning and the end of the REM sleep episode were scored according to the rules by Lapierre and Montplaisir^10^.

### RWA analysis

EMG activity during REM sleep was quantified visually according to the SINBAR criteria for phasic and tonic EMG activity in the mentalis and FDS muscles. All artifacts and increases in EMG tone due to arousals from respiratory events were excluded from the quantitative scoring before the analysis of EMG activity. Consequently, the SINBAR score, which is the percentage of REM sleep with tonic or phasic mentalis EMG activity and phasic FDS EMG activity, was calculated^12^. Indices of tonic RWA and phasic RWA were assessed according to SINBAR scoring criteria, and mixed EMG RWA (simultaneous tonic and phasic RWA)^13^ were also calculated and analyzed separately.

### MRI examination and processing

MRI examination was performed on a 3T scanner (Siemens Skyra 3T, Siemens Healthcare, Erlangen, Germany) with a 32-channel head coil. The protocol included axial 3D T1-weighted Magnetization Prepared Rapid Gradient Echo (MPRAGE, repetition time (TR) 2200 ms; echo time (TE) 2.4 ms; inversion time (TI) 900 ms; flip angle (FA) 8°; field of view (FOV) 230 × 197 × 176 mm; voxel resolution 1.0 × 1.0 × 1.0 mm^3^) and multi-echo gradient recalled echo (GRE; TR = 34 ms; six equispaced echoes between 4.9 and 29.5 ms; FA = 15°; FOV = 192 × 256 × 128 mm^3^; voxel resolution = 0.57 × 0.57 × 2 mm^3^) pulse sequences.

GRE phase images from all 32 channels (each corresponding to a separate radiofrequency coil) were combined offline using the ASPIRE (A Simple Phase Image Reconstruction for multi-Echo data) Method^76^. QSM was reconstructed from the brain stripped GRE magnitude and phase images with the QSMbox software package for MATLAB (https://gitlab.com/acostaj/QSMbox) using the previously validated multi-scale dipole inversion algorithm^77^. We used the default pipeline for coil-combined multi-gradient echo data and the process involved re-slicing the complex GRE data to an isotropic (i.e. 0.57 × 0.57 × 0.57 mm^3^) voxel resolution via zero-padding.

**Fig. 4:**
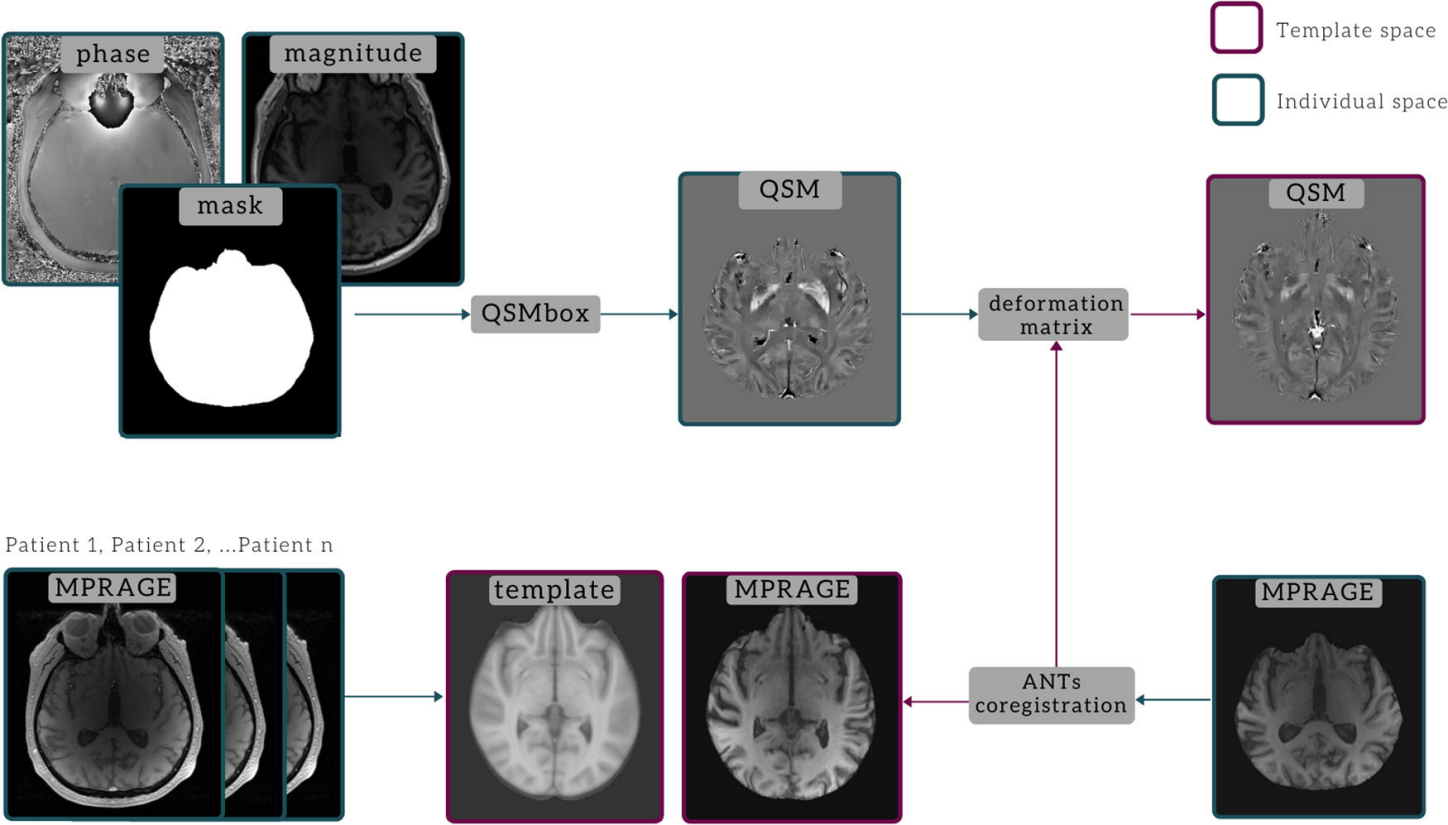
Schematic illustration of MRI data processing pipeline for QSM reconstruction and spatial normalization. ANTs advanced normalization tools, QSM quantitative susceptibility mapping, MPRAGE Magnetization Prepared Rapid Gradient Echo.

MPRAGE images were co-registered to the first-echo GRE magnitude image and were thus spatially aligned with QSM images. A study-specific T1 template was created using the advanced normalization tools (ANTs) software (http://stnava.github.io/ANTs) by averaging the MPRAGE images of 15 randomly selected iRBD patients. The use of customized template was previously recommended for optimized VBM^78^. The MPRAGE images of each subject were normalized to the space of this template using the ANTs software. The deformation matrix obtained in this step was applied to the QSM images, bringing them to the template space (Fig. 4). Normalized QSM images were smoothed using a Gaussian kernel with a full-width at half maximum of 2 mm before entering the voxel-wise group analysis. The small smoothing kernel was chosen to prevent the increase of the blurring bias of small structures^79^.

### Statistical analysis

Voxel-wise statistical analyses of QSM images were performed in the Statistical Parametric Mapping (SPM12, version 7771) toolbox (https://www.fil.ion.ucl.ac.uk/spm) running under MATLAB (version 2022a) after the application of a hand-drawn explicit mask. Three masks were applied separately at the brainstem according to the hypothesis driven area of interest: 1) mesencephalon and ponto-mesencephalic border, 2) dorsal pons and 3) ventral medulla. The analyses were performed independently within each mask. The between-group comparison was performed using a general linear model with age and gender as regressors of no interest.

Associations of magnetic susceptibility and RWA parameters were analyzed using a multiple linear regression design with age and gender as covariates of no interest. Threshold free cluster enhancement (TFCE) in SPM12 (http://www.neuro.uni-jena.de/tfce), set to default parameters (*E* = 0.5), with *p*-value *p* < 0.05, corrected for family wise error (FWE) was used for cluster permutation analysis based on each design matrix.

Afterwards the T1 template was normalized to standard MNI125 space (Montreal Neurological Institute, McGill University, Canada) using SPM12, then the warp was used to normalize the statistical maps and the QSM template as well. This step enabled the identification of the affected brainstem structures, using an open access interactive atlas Brainstem Navigator (https://www.nitrc.org/projects/brainstemnavig)^80^ and a Topographic Atlas of the Human Brainstem in the Ponto-Mesencephalic Junction Plane^81^.

The statistical analysis of clinical and polysomnographic variables was performed using the JASP software. Categorical variables were analyzed using a Fisher’s exact test. For the continuous variables, a Shapiro–Wilk test was used for testing the correspondence of the calculated parameters to a normal distribution. A Mann–Whitney U test was used for the inter-group comparison. Statistical significance was defined as *p* < 0.05. The Holm–Bonferroni method was applied to correct the family-wise error on the level of the continuous variables.

### Reporting summary

Further information on research design is available in the Nature Research Reporting Summary linked to this article.

## Supplementary information

Reporting Summary

## Data Availability

The datasets used and analyzed during the current study are available from the corresponding author on request.
